# Symmetry of root and root canal morphology of mandibular incisors: A cone-beam computed tomography study *in vivo*

**DOI:** 10.4317/jced.55629

**Published:** 2019-06-01

**Authors:** Federico Valenti-Obino, Dario Di Nardo, Linda Quero, Gabriele Miccoli, Gianluca Gambarini, Luca Testarelli, Massimo Galli

**Affiliations:** 1 Department of Oral and Maxillo Facial Sciences “Sapienza” University of Rome, Italy

## Abstract

**Background:**

Aim of this study was to analyze the root canal configuration in mandibular central and lateral incisors in vivo using cone-beam computed tomography (CBCT) imaging.

**Material and Methods:**

A total of 487 mandibular central incisors and 491 mandibular lateral incisors from 250 patients were examined using CBCT imaging, previously taken for diagnosis and treatment. The number of roots, root canal system configuration, presence of apical confluences, distance between confluences and radiographic root end, symmetry between left and right elements were recorded and statistically analyzed.

**Results:**

All the examined teeth presented only one root. No significant differences were found between the prevalence of two root canals in mandibular central incisors (219 teeth, 45%) compared to lateral incisors (211 teeth, 43%).

**Conclusions:**

The percentage of Vertucci type II configuration was higher than expected, being more frequent than type III. Type I was the prevalent, while other configurations were present but rare.

** Key words:**Cone-beam computed tomography, mandibular incisors, root canal anatomy, confluences.

## Introduction

The study of the anatomy is very important in endodontics, since most of the errors that occur during an endodontic treatment are related to failure in respecting the canal anatomy (1); the existence of an untreated canal may be a reason of failure (2). To achieve these goals, clinical examination and diagnostic imaging are both essential elements of preoperative diagnosis and treatment planning (3). Ideally, internal anatomical complexities, such as the number of canals, their shapes and trajectories, including the presence of confluences and bifurcations should be assessed before undertaking instrumentation.

The study of endodontic anatomy can be divided into *ex vivo* methods, performed on extracted teeth, and *in vivo* methods, performed on patients (4). Amongst these various methods, CBCT recently become the most valuable tool for researching endodontic anatomy *in vivo*, because it is a non-invasive tool that can provide images displayed in coronal, sagittal and axial planes. Moreover, it defines precisely the position of the tooth and allow studies about symmetry (5).

Tooth configurations are usually classified according to Vertucci and the great majority of studies used this classification; more recently Ahmed and Dummer (6) proposed a new classification. It is a more accurate method, based on in vitro microCT evaluations of extracted teeth. Therefore it requires higher resolution of images which is not always achievable by *in vivo* CBCT to avoid unnecessary high radiation doses to the patient.

The aim of the present study was to analyze *in vivo* the root canal configuration in mandibular central and lateral incisors using CBCT and Vertucci classification, to assess clinically relevant anatomical features.

## Material and Methods

Sample selection: A total of 487 mandibular central incisors and 491 mandibular lateral incisors were examined. Teeth were selected from the CBCT examinations of 250 patients (130 males and 120 females) with an age ranging between 18 and 79 years. Images were obtained from CBCT examinations as part of diagnosis and treatment planning of patients who required large field of view for other reasons. The research was approved by the Ethical Committee of Policlinico Umberto I, Rome, Italy (ref. 582/17).

The samples were selected according to the following criteria:

• available CBCT images of lower incisors with complete root formation;

• absence of root canal treatment, crowns and posts;

• CBCT images without scattering.

Teeth with immature apexes and root resorption were excluded.

Image acquisition: CBCT images had been taken using the GXDP-500 system (Gendex Dental, Biberach, Germany), operating at 90 kVp and 7 mA, with an exposure time of 23 s and a voxel size of 0.2 mm3, with a field of view of 13x9x13 cm, with an estimated dose of about 5 mSv, allowing measurements to an accuracy of 0.2 mm.

Image evaluation: Through the use of Horos™ software (The Horos Project, 64-bit medical image viewer, GNU Lesser General Public Licence, version 3.0) three-dimensional reconstructions were analyzed to evaluate the parameters of interest. Images were reworked according to the axial, sagittal and coronal planes. CBCT images were viewed on reconstructions according to the axial plane, scrolling the cursor in the coronal-apical direction before, and then in the apical-coronal direction, to get a detailed view of the root canal system of examined teeth. This action was repeated 3 times, and when the images in the axial plane were not clear, the tooth was also inspected in the three-dimensional reconstruction. The software had a specific tool which allowed precise measurements (~0,01 mm).

The following parameters were evaluated: number of roots, root canal system configuration, presence of apical confluences, distance between confluences and radiographic root end, and symmetry between left and right elements in the same individual.

**Figure 1:**
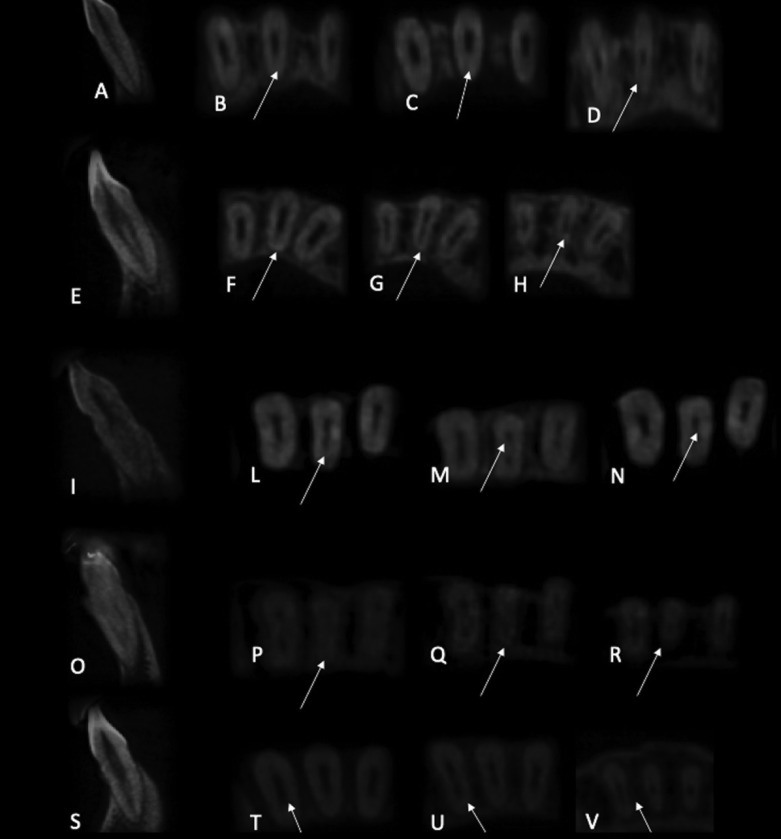
Sagittal plane of CBCT scanning Type I (A), Type II (E), Type III (I), Type IV (O), Type VII (S). Axial plane of CBCT scanning in the coronal, middle, and apical thirds of the root displayed variations in canal morphology: (B-D) Type I, (F-H) Type II, (L-N) Type III, (P-R) Type IV, (T-V) Type VII.

The classification of the canal morphology was done according to the Vertucci’s criteria (Fig. 1):

• Type I: single canal from the pulp chamber to the apex;

• Type II: two different canals emerge from the pulp chamber but converge to the apex;

• Type III: a canal emerges from the pulp chamber, divides into two within the root and emerges into one at the apex;

• Type IV: two different canals from the pulp chamber to the apex;

• Type V: a single canal emerge from the pulp chamber and divides into two at the apex;

• Type VI: 2 different canals emerge from the pulp chamber, join at the middle of the root and then divide again into two with two different apical foramina;

• Type VII: one canal in the pulp chamber that divides into two and rejoins within the root, and redivides into two canals at the apex;

• Type VIII: 3 separate canals from the pulp chamber to the apex.

Statistical Analysis: The results were analysed statistically using SPSS 20.0 (SPSS, Inc., Chicago, IL, USA) with the significance set at *p*<0.05. One-way ANOVA was used for the association between the variables along with the post hoc tests, Tukey’s HSD and Games–Howell. The t-test was used to compare the mean distances from confluence to radiographic root end.

## Results

Number of roots and canal system configuration ( Table 1) according to Vertucci ( Table 2):

**Table 1:**
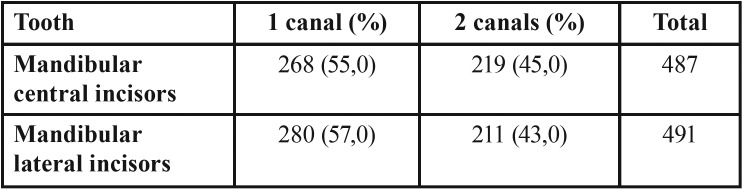
Prevalence of the number of root canals in mandibular incisors.

**Table 2:**
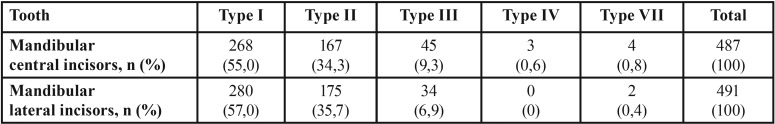
Distribution of root canal types according to vertucci classification in mandibular incisors.

• All examinated teeth presented only one root.

• For mandibular central incisors type I Vertucci configuration was present in 55% of cases, type II Vertucci configuration in 34,3% of cases, type III Vertucci configuration in 9,3% of cases, type VII Vertucci configuration in 0,8% of cases and type IV Vertucci configuration in 0,6% of cases.

• For mandibular lateral incisors type I Vertucci configuration was present in 57% of cases, type II Vertucci configuration in 35,7% of cases, type III Vertucci configuration in 6,9% of cases, type VII Vertucci configuration in 0,4% of cases.

• No statistical differences were found between central and lateral incisors (p<0,05), except for the lack of type IV configuration in lateral incisors.

Apical confluences:

• Apical confluences were present in all the incisors with a type II, type III and type VII Vertucci configuration. Overall there was the 44,4% (216) of confluences in central incisors and 43% (211) in lateral incisors.

• No statistical differences were found between central and lateral incisors (*p*<0,05).

Distance between confluences and radiographic root end:

• The average distance between confluences and radiographic root end in mandibular central incisors was 3,363 mm (+/-0,2) in type II Vertucci configuration and 3,391mm (+/-0,2) in type III Vertucci configuration. In mandibular lateral incisors was 3,606mm (+/-0,2) in type II Vertucci configuration and 3,498mm (+/-0,2) in type III Vertucci configuration. The distance in type VII was 3,258mm (+/-0,3) in central incisors and 2,831mm (+/-0,3) in lateral incisors.

• No statistical differences were found between central and lateral incisors, except for type VII (*P*<0,05).

Symmetry of root canal morphology between left and right elements of the same individual:

• Symmetry was found in 44,6% of cases in mandibular central incisors and in 44,8% of cases in mandibular lateral incisors.

• No statistical differences were found between central and lateral incisors (*p*<0,05).

## Discussion

In literature, multiple methods have been used to investigate root canal anatomy (7), including canal staining and clearing techniques (8,9), dentin troughing under magnification (10), scanning electron microscope (11,12), micro-computed tomography (micro CT) (13,14), magnetic resonance (15-16), ultrasonics(17), serial cross-sectioning (18), radiographic examination (19,20) and CBCT (21-23). CBCT(24) allows *in vivo* 3D evaluations of canals: it overcomes the limitations of conventional radiography because it reduces the superimposition of the surrounding structures, it also allows to study a greater number of teeth defining symmetry between left and right in the same patient (25).

Many articles (26-34) about endodontic anatomy of mandibular incisors are present in literature, showing different possible configurations. Most of these researches showed that in mandibular incisors the prevalent configuration is Vertucci type I (only one canal), with a percentage ranging from 96,2% to 32,5% (mean value 64,3%). These contradictory data could be explained by the different methodologies adopted ( Table 3), since only some studies were conducted with CBCT (35-43), and very few examined other clinically relevant parameters like confluences (44) and symmetry (45). Theoretically, there should be no significant differences between studies using CBCT or extracted teeth. The main differences could derive from samples size, population and age. The last two parameters, however, could be difficult to assess in an *in vitro* study, because origin and position of the extracted tooth could be unknown.

**Table 3:**
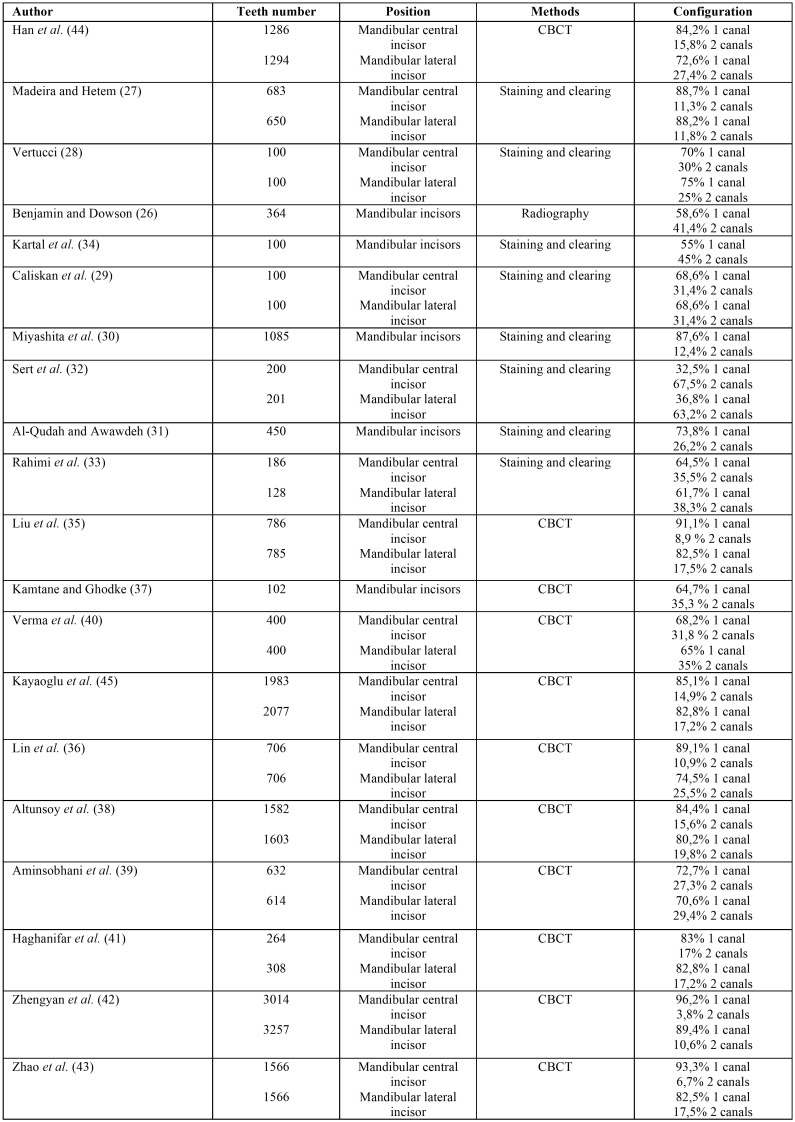
Percetanges of root canal types found in mandibulat anterior teeth in previous studies.

In the present study the following parameters were evaluated: number of roots, root canal system configuration, presence of apical confluences, distance between confluences and radiographic root end, symmetry between left and right elements, based on author’s previous CBCT studies on molars (5,7). In these study the clinical relevance of confluences (one of the major cause of rotary instruments’ separation) was highlighted. Data about symmetry could be clinically helpful for dentists using only traditional, 2D radiographs for initial case assessment.

Our results showed a high prevalence of type II configuration in mandibular lateral incisors: it was found in 35.7% of the teeth examined. This result was lower than the study by Benjamin and Dawson (26). On the contrary, the majority of studies (27-40) performed on the mandibular lateral incisors, showed a significantly lower prevalence, ranging from 1% to 26,9%.

Similar results were found in the mandibular central incisors, with a 34,3% of prevalence of type II. These findings were higher than all the studies previously performed (27-40), but lower than the study by Benjamin and Dawson (26), ranging from 0,1% to 27,5%.

Type I Vertucci configuration was present in 55% of mandibular central incisors, lower than other studies (26-31,35-44), but in accordance with Kartal *et al.* (34), and superior to Sert *et al.* (32). Similar comparisons were found for lateral incisors, showing a 57% prevalence. The total range was from 89,4% to 36,8%.

Type III Vertucci configuration was found in the 9,3% of mandibular central incisors and 6.9% of lateral incisors. These findings were different from the majorities of previous researches (26-40), which showed an average percentage ranging from 0% to 27%.

Type IV configuration was found in 0,6% of mandibular central incisors and totally absent in mandibular lateral incisors, lower than previous studies (26-28,30-40), with a range from 0% to 5,9%.

Type VII configuration was found in 0,8% of mandibular central incisors and in 0,4% of mandibular lateral incisors; interestingly, only the research by Han *et al.* (44) ever showed this configuration (0,39% in mandibular central incisors and 0,08% in mandibular lateral incisors).

The mean distance between confluences in type II mandibular central incisors was 3,363 mm (ranging from 1,326 mm to 5,884 mm), and in lateral incisors was 3,606 mm (ranging from 1,442 mm to 6,432 mm), showing no significant differences between the two groups of teeth. Similarly, for the type III configuration in mandibular central incisors mean distance was 3,391 mm (ranging from 1,130 mm to 6,001 mm) while in lateral incisors was 3,498 mm (ranging from 1,429 mm to 6,231 mm). These results were in accordance with previous studies (5) performed on confluences in molars, and significantly lower than the research of Han *et al.* (44), performed on mandibular incisors, where the distribution was concentrated in the 6-12 mm range. The mean distance in type VII was 3,258 mm (ranging from 0,657 mm to 4,671 mm) in central incisors and 2,831 mm (ranging from 2,648 mm to 3,014 mm) in lateral incisors.

Only a single study previously reported the localization of confluences in lower incisors (44). In the present study results were very different, since they were mostly found in the apical third, 3-4 mm shorter than radiographic root end.

When analyzed in the same individual, the symmetry was present in the 44,6% of the mandibular central incisors and the 44,8% of the mandibular lateral incisors, with no statistical differences between the two groups. Our results were similar with the research by Kayaoglu *et al.* (45) and lower than other studies performed on molars (5).

## Conclusions

The percentage of mandibular incisors presenting a complex anatomy (two canals with different configurations) is higher than previously reported.

These results highlighted that only an accurate preoperative radiographic exam could reveal and identify complex canal configuration in mandibular incisors.
